# Using local chromatin structure to improve CRISPR/Cas9 efficiency in zebrafish

**DOI:** 10.1371/journal.pone.0182528

**Published:** 2017-08-11

**Authors:** Yunru Chen, Shiyang Zeng, Ruikun Hu, Xiangxiu Wang, Weilai Huang, Jiangfang Liu, Luying Wang, Guifen Liu, Ying Cao, Yong Zhang

**Affiliations:** 1 Translational Medical Center for Stem Cell Therapy & Institute for Regenerative Medicine, Shanghai East Hospital, School of Life Science and Technology, Tongji University, Shanghai, China; 2 Shanghai Key Laboratory of Signaling and Disease Research, Tongji University, Shanghai, China; National University of Singapore, SINGAPORE

## Abstract

Although the CRISPR/Cas9 has been successfully applied in zebrafish, considerable variations in efficiency have been observed for different gRNAs. The workload and cost of zebrafish mutant screening is largely dependent on the mutation rate of injected embryos; therefore, selecting more effective gRNAs is especially important for zebrafish mutant construction. Besides the sequence features, local chromatin structures may have effects on CRISPR/Cas9 efficiency, which remain largely unexplored. In the only related study in zebrafish, nucleosome organization was not found to have an effect on CRISPR/Cas9 efficiency, which is inconsistent with recent studies in vitro and in mammalian cell lines. To understand the effects of local chromatin structure on CRISPR/Cas9 efficiency in zebrafish, we first determined that CRISPR/Cas9 introduced genome editing mainly before the dome stage. Based on this observation, we reanalyzed our published nucleosome organization profiles and generated chromatin accessibility profiles in the 256-cell and dome stages using ATAC-seq technology. Our study demonstrated that chromatin accessibility showed positive correlation with CRISPR/Cas9 efficiency, but we did not observe a clear correlation between nucleosome organization and CRISPR/Cas9 efficiency. We constructed an online database for zebrafish gRNA selection based on local chromatin structure features that could prove beneficial to zebrafish homozygous mutant construction via CRISPR/Cas9.

## Introduction

Zebrafish is a widely used model organism in basic biological and clinical research, and many mutants have been constructed with phenotypes similar to human diseases [1, 2]. The workload and cost of zebrafish mutant screening is largely dependent on the mutation rate of the founder (F_0_). When the mutation rate of F_0_ progeny is high enough (in practice, higher than 30%), homozygous or compound heterozygous mutants can be obtained through inbreeding within F_0_ (termed an incross). Otherwise, an additional generation F1, generated by outcrossing F_0_ with the wild type and screening for heterozygous offsprings, is required to obtain homozygous or compound heterozygous mutants. When the mutation rate of F_0_ is very low (in practice, lower than 15%), it would be very time- and labor-consuming to obtain homozygous mutants. Recently, the CRISPR/Cas9 system, which is a powerful and effective genome editing technology, has been successfully applied to zebrafish studies for identifying genes involved in biological processes [3–5], disease model construction [6], drug screening [7], and lineage tracing [8]. By co-injecting guide RNA (gRNA) and Cas9-encoding mRNA in zebrafish one-cell stage embryos, the CRISPR/Cas9 efficiency can reach over a high level for some certain gRNAs [9]. However, CRISPR/Cas9 efficiencies vary greatly for different gRNAs, even for those targeting the same genes (S1A and S1B Fig). Therefore, selecting more effective gRNAs can dramatically improve the CRISPR/Cas9 efficiency, which is especially important for zebrafish mutant construction.

Sequence features of gRNA targets and neighboring regions have been shown to influence CRISPR/Cas9 efficiency [10–12]. For effective gRNAs, guanines are preferred at -1 and -2 upstream of the protospacer-adjacent motif (PAM motif), whereas thymine is disfavored at -4 proximal to the PAM. Cytosine is preferred at -3 downstream of the Cas9 cutting site [13]. Although the above sequence features obtained from studies in mammalian cell lines are also effective for gRNA target selection in zebrafish (S1C Fig), we did not observe a correlation between scores for measuring those gRNA sequence features and CRISPR/Cas9 efficiency (S1D Fig). A recent systematic study in zebrafish showed that target sequence features have effects on gRNA stability, which in turn correlated with CRISPR/Cas9 efficiency [14].

Besides the influence of sequence features, local chromatin structures may have effects on CRISPR/Cas9 efficiency in zebrafish. Generally, local chromatin structure includes two aspects, nucleosome organization and chromatin accessibility. For nucleosome organization, a recent *in vitro* experiment showed that the Cas9 efficiency was strongly inhibited when the PAM motif was located within a well-positioned nucleosome region [15]. Recent studies in mammalian cells showed that nucleosome works as an inhibitor to decrease Cas9 activity [16, 17]. For chromatin accessibility, studies in mammalian cell lines have shown that the openness of chromatin is important for determining Cas9 efficiency [18] and potential Cas9 off-target binding sites are enriched in open chromatin regions, indicating that open chromatin regions are more likely to be bound by Cas9 [19]. The abovementioned studies indicated the potential usefulness of local chromatin structure information in optimizing gRNA selection. However, the effects of local chromatin structure on CRISPR/Cas9 efficiency in zebrafish were largely unexplored. In the only related study in zebrafish, a comparison was performed between gRNA activity and nucleosome organization profiles [20]; however, the effects of nucleosome organization on CRISPR/Cas9 efficiency were not observed [14], which was inconsistent with the recent studies *in vitro* and in mammalian cells [15–17].

There are two obstacles to studying the effects of local chromatin structure on CRISPR/Cas9 efficiency in zebrafish. First, compared with mammalian cell lines, the local chromatin structures of zebrafish are highly dynamic during early embryogenesis [20], which increases the difficulty of linking the CRISPR/Cas9 efficiency with local chromatin structure at a certain developmental stage. Second, the high-throughput local chromatin structure profiles are limited in early zebrafish embryo; for example, chromatin accessibility profiles for this process are not currently available. To identify the effects of local chromatin structure on CRISPR/Cas9 efficiency, we first determined that the majority of genome editing events of the CRISPR/Cas9 system occurred before the dome stage. Accordingly, we reanalyzed our published nucleosome organization profiles in the 256-cell and dome stages and then generated chromatin accessibility profiles in those two stages using ATAC-seq technology. Our study demonstrated that chromatin accessibility (measured by ATAC-seq signals) showed positive correlation with CRISPR/Cas9 efficiency, but we did not observe a clear correlation between nucleosome organization (measured by MNase-seq signals) and CRISPR/Cas9 efficiency. We also provided a webserver for zebrafish gRNA selection based on chromatin accessibility features that could prove beneficial for gRNA selection in genome editing based on the CRISPR/Cas9 system.

## Materials and methods

### Study approval and zebrafish maintenance

Zebrafish were maintained according to the standard protocol[21]. All zebrafish lines used in this study were maintained in accordance with institutional guidelines on animal usage and maintenance of Tongji University, under a protocol approved by Institutional Animal Care and Use Committee of Tongji University. Zebrafish embryos for injection were obtained via natural mating of wild-type TU-strain zebrafish (6–12 months).

### gRNA design and template synthesis

We produced target sets for which the mutation rates were detected by high-throughput sequencing. For the mutation rates of the targets detected by high-throughput sequencing (NGS data), 20 bp target candidates were screened from the whole genome exon followed by the PAM sequence. In addition, other locations in the PCR product should not have this restriction site. To rule out the influence of target non-fidelity, we chose gRNA with a score of 90+ (calculated at http://crispr.mit.edu/). To improve the transcription efficiency of gRNA in vitro, “GG” was added to the 5’ end of most targets. We employed a two-oligo PCR method to synthesize the template DNA required for the in vitro transcription. The forward primer sequences included three types: 5’-AATTAATACGACTCACTATA (0-2G) (N20) GTTTTAGAGCTAGAAATAGC-3’, 5’-GAAATTAATACGACTCACTATA (0-2G) (N20) GTTTTAGAGCTAGAAATAGC-3’ and 5’-TAATACGACTCACTATA (0-2G) (N20) GTTTTAGAGCTAGAAATAGC-3’. The scaffold oligo sequence was 5’GATCCGCACCGACTCGGTGCCACTTTTTCAAGTTGATAACGGACTAGCCTTATTTTAACTTGCTATTTCTAGCTCTAAAAC-3’. The PCR reaction system included 37 μl ddH2O, 5 μl 10×Pfu buffer, 4 μl 2.5 mM dNTPs, 1 μl forward primer (100 mM), 1 μl reverse primer (100 mM), and 2 μl Pfu DNA polymerase. The PCR condition was programmed as follows: 98°C for 30 s; 30 cycles of 98°C for 10 s, 60°C for 30 s, and 72°C for 15 s; and 72°C for 10 min. The PCR products were column purified (QIAquick PCR Purification Kit, QIAGEN, 28104) and titrated by a NanoDrop 2000 system.

### Preparation of gRNA and Cas9 mRNA

For the synthesis of gRNA, 1 μg template was used in transcription in vitro with a half reaction system (MEGAshortscript T7, Life Technologies, AM1354). The product was purified by phenol-chloroform and ethanol precipitation and stored at -80°C.

For the synthesis of Cas9 mRNA, the pGH-T7-zCas9 plasmid was used for transcription of Cas9 mRNA. The plasmid was linearized using XbaI (New England BioLabs, R0145S) and column purified (QIAquick PCR Purification Kit, QIAGEN, 28104). We used 1 μg linearized plasmid for transcription in vitro (mMESSAGE mMACHINE T7, Life Technologies, AM1344). For Cas9 mRNA used in multiplexing co-injection of 6–7 gRNAs, a 40 μl reaction system was used, and the products were column purified (Zymo Research, R1016) to increase transcript production and meet the concentration requirement. For Cas9 mRNA used in co-injecting of 1–2 gRNAs, the RNA products were purified using a kit (RNeasy MiNi Kit, QIAGEN, 74104). All the products were titrated with a NanoDrop 2000 system.

### Microinjection of gRNA and Cas9 mRNA

For the NGS data, we injected 6 to 7 gRNAs and Cas9 mRNA mixture into the egg yolk at one-cell stage. After mixing, the final concentration of each gRNA and Cas9 mRNA is 12.5 ng/ul and 150 ng/ul, respectively. For pairwise experiments, we co-inject two gRNAs and Cas9 mRNA mixture into the egg yolk at once at one-cell stage. In each target pair, the final concentration of each gRNA and Cas9 mRNA is 75 ng/ul and 500ng/ul, respectively. For all microinjections, 1nl gRNA/Cas9 mixture was injected into the yolk at the one-cell stage. All embryos were cultured at 28.5°C after injection.

### Time-course experiment

We selected 4 target sites (*smc1a*, *tarbp1*, *vps13c*, *ccdc129*) with mutation rate >30% as detected by restriction enzyme digestion. For each target, gRNA was co-injected with Cas9 mRNA into the egg yolk at final concentrations of 80 ng/ul and 150 ng/ul, respectively, at one-cell stage. 1nl gRNA/Cas9 was used for all injections. Embryos of each stage were collected at six developmental stages: 64 cell (2 hours post fertilization, hpf, 120 embryos), 256 cell (2.5 hpf, 30 embryos), dome (4.33 hpf, 20 embryos), bud (10 hpf, 10 embryos), 24 hpf (10 embryos) and 48 hpf (10 embryos). For each stage, all embryos were lysed in 190 μl lysis buffer (100 mM Tris-HCl, pH 8.3; 200 mM NaCl; 0.4% SDS; 5 mM EDTA) and 10 μl Proteinase K (20 ng/μl) and incubated at 56°C overnight. The genomic sequences of target locus were PCR amplified from DNA lysate of embryos. The mutation rate was detected by restriction enzyme digestion and measured by ImageJ. For each target, the experiments were repeated three times.

### Genome extraction and target PCR

For both the NGS data, 10 embryos were collected at 24 hour post-fertilization (hpf) and then lysed in 190 μl lysis buffer (100 mM Tris-HCl, pH 8.3; 200 mM NaCl; 0.4% SDS; 5 mM EDTA) and 10 μl Proteinase K (20 ng/μl) and incubated at 56°C overnight. Target sequences were PCR amplified by LA Taq (LA Taq DNA Polymerase, Takara, RR002A).

### NGS preparation and sequencing

For the NGS data, we designed reverse primers approximately 30~70 bp away from the 3’ end of the 20 bp target to ensure the accuracy of the sequencing. The product length was approximately 180 bp to 250 bp. The primers were specific for each target site, and 110 targets were divided into two groups to construct an Illumina sequencing library using the ThruPLEX DNA-seq Kit (RUBICON). Products were cleaned by Agencourt AMPure XP beads (Beckman, A63881) and then sequenced using an Illumina HiSeq X Ten sequencer.

### ATAC-seq experiment

The ATAC-seq of the zebrafish embryos was a minor modification of the original method [22]. Briefly, 256-cell and dome stage embryos (approximately 10,000 cells) were harvested and washed 3 times with pre-iced PBS. Then, the embryos were re-suspended in cold lysis buffer (10 mM Tris-HCL, pH 7.4, 10 mM NaCl, 3 mM MgCl_2_, 0.1% IGEPAL CA-630), lysed by pipetting up and down 5–10 times using a widened pipet, and kept on ice for 5 minutes. The isolated nuclei were then pelleted and re-suspended in the transposition reaction (25μl 2_TD Buffer, 2.5μl Tn5 transposes, 22.5μl nuclease-free H2O) for 30 minutes at 37°C, and then column purified (MinElute PCR Purification Kit, QIAGEN, 28004). The PCR was performed to yield the libraries by adding two different barcodes. The PCR conditions were as follows: 72°C for 5 min, 98°C for 30 s; 15 cycles of 98°C for 10 s, 63°C for 30 s, and 72°C for 1 min; and hold at 4°C. Then, the libraries were purified with Agencourt AMPure XP beads and sequenced by an Illumina HiSeq X Ten sequencer.

### Cas9 datasets involved

We divided our experimental Cas9 targets into two groups with cleavage efficiencies that were detected by the RE and next generation sequencing (NGS), respectively. Another NGS dataset (SRP059430) was also involved in this study.

### Cas9 cleavage efficiency calculation from NGS data

Both groups of NGS detection data were processed as follows. According to the unique forward and reverse primers of each designed target, all sequenced reads were divided into fastq files, which contained only reads from target regions as expected. Then, paired reads of each targets were mapped back to the zebrafish genome (zv9/danRer7 assembly) using the bwa (v 0.7.10) [23] mem command with default parameters (see S1 File for mapping summary details). A reads pair was kept when both reads were mapped back to the genomic location around designed target site. Subsequently, for each reads, a custom Python code was used to calculate insertion or deletion (indel) status around designed cutting sites by analyzing the CIGAR string. All indel events and their supporting reads counts were summarized. Then low coverage indel events (i.e. occurrence rate less than 0.5%) were filtered out to avoid the influence of sequence or PCR errors. Finally, for each target site, we have two numbers: the number of reads supporting any indel events, and the total number of reads covering the 40 bp region around the designed Cas9 cutting site (i.e. 3 bp upstream the PAM sequence). The CRISPR/Cas9 efficiency was defined as the ratio of the above two numbers.

### Nucleosome linker and nucleosome occupied region identification

MNase-seq data of the 256-cell and dome stage were obtained from a previous study (GSE44269). Because our study focused on only Cas9 targets on coding regions, the reads mapped on intergenic regions were excluded to save calculation time. To identify the nucleosome midpoints, 73 bp was added to the 5’ end of the coordinate for each mapped read. Then, all nucleosome midpoints in two stages were combined to predict stable nucleosomes using GeneTrack (with parameters -e 147 -s 20) [24]. Among all nucleosome peaks predicted by GeneTrack, we removed those with low enrichment scores and merged the remaining peaks, with those presenting lengths < 180 bp identified as nucleosome occupied regions. The other merged peaks were regarded as dynamic nucleosome regions within the 256-cell and dome stage. Subsequently, the complementary regions of merged peaks longer than 10 bp and shorter than 100 bp were identified as nucleosome linker regions. In summary, zebrafish genome region was divided into nucleosome linker (NL) regions, nucleosome occupied (NO) regions and nucleosome dynamic regions.

### Open chromatin and close chromatin region identification

The ATAC-seq reads of 256-cell and dome stages were aligned to the zebrafish genome built using zv9/danRer7 using bowtie2 (v 2.2.3) [25]. We removed the reads that were mapped to the scaffold chromosome and the reads with MAPQ < 30. The remaining reads were used to call peaks using the MACS2 (v 2.0.10.20131028) [26] callpeak function with parameter -q 0.01 –SPMR (see S2 File for ATAC-seq data summary). We combined the peaks called from the two stages. Each peak summit was extended 1000 bp upstream and 1000 bp downstream, and the merged regions were identified as open chromatin (OC) regions. The remaining regions on the genome were regarded as close chromatin regions (CC) regions.

### Web-server establishment

We scanned the entire zebrafish exome (zv9 assembly, Refseq annotation) and obtained 2,922,312 CRISPR/Cas9 potential targets by recognizing distinct PAM (NGG) motif. According to the identification of chromatin structure (OC/CC) regions, all Cas9 targets on the zebrafish exome were divided into OC/CC regions, respectively. For sequence feature evaluation, we used the source code of Sequence Scan for CRISPR (SSC, http://cistrome.org/SSC) [13] to calculate the scores for each target. Subsequently, for each potential target, we evaluated the possibility of off-target binding by calculating 0–2 mismatched sites on the zebrafish genome. We used bowtie version 1.0.1 [27] to output all 0–2 mismatched sites on the genome with -v 2 -a parameters, and then a custom Python code was used to calculate the mismatched site number for each target. Taken together, each target had three features that described the chromatin accessibility status, sequence feature evaluation and off-target potential. We integrated this information and produced a web-sever that can be used to easily obtain gRNA targets for zebrafish exomes by querying nucleotide sequences or gene names to optimize the design of genome editing experiments based on the CRISPR/Cas9 system.

### Data access

CRISPR/Cas9 gRNA target sites PCR amplification products sequencing data and ATAC-seq data reported in this paper have been submitted to the Genome Sequence Archive of BIG Data Center, Beijing institute of Genomics (BIG), Chinese Academy of Sciences (http://gsa.big.ac.cn/) with accession number PRJCA000283.

## Results

### CRISPR/Cas9 introduces mutations mainly before dome stages

**Fig 1:**
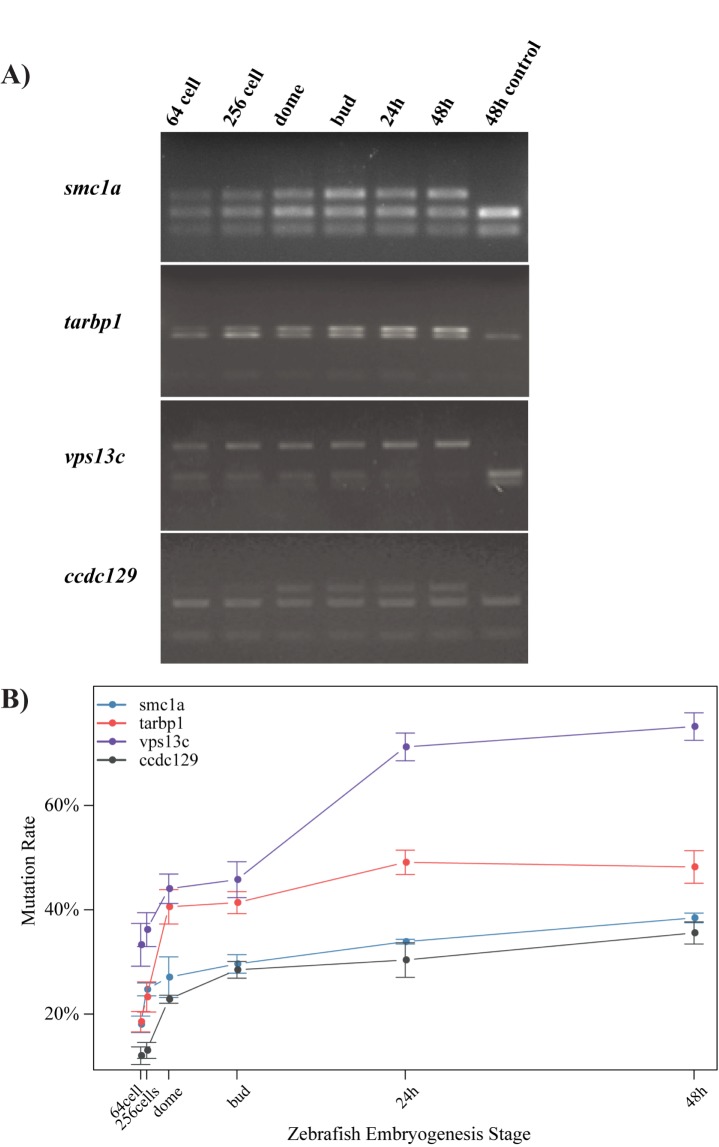
CRISPR/Cas9 mainly generated mutation before dome stage in zebrafish. gRNAs targeting on 4 genes (*smc1a*, *tarbp1*, *vps13c*, *ccdc129*) were injected into the one-cell embryos of zebrafish, and mutation events were detected by using restriction enzymes (RE) at following embryogenesis stages: 64-cell (2 hpf), 256-cell (2.5 hpf), 1k-cell (3 hpf), dome (4.33 hpf), bud (10 hpf), 24 hpf and 48 hpf, a 48 hpf control was included. **(A)** PCR products of each Cas9 target were treated by specific RE. **(B)** Fluorescence intensity was transferred to mutation rates by ImageJ. Blue, red, purple and grey line represent mutation rate of gRNAs targeting on *smc1a*, *tarbp1*, *vps13c* and *ccdc129*, respectively. X-axis was arranged according to hpf time course of zebrafish embryo development. Error bars were calculated from replicates of gRNAs targeting on each gene.

To investigate the correlation between the local chromatin structure and CRISPR/Cas9 efficiency, the developmental stages during which CRISPR/Cas9 causes mutations in zebrafish must be identified because the local chromatin structure is highly dynamic during this process. We selected four gRNAs targeting at 4 genes (*smc1a*, *tarbp1*, *vps13c*, *ccdc129*), respectively, with high CRISPR/Cas9 efficiencies and examined the mutation rates of their targets at the following six developmental stages: 64 cell (2 hours post fertilization, hpf), 256 cell (2.5 hpf), dome (4.33 hpf), bud (10 hpf), 24 hpf and 48 hpf (See S3 File for target information). The mutation rates of the targets were measured via digestion with restriction enzymes (REs). As shown in Fig 1, despite the gRNA of *vps13c* showed continuous dramatic increasing activity even after dome stage, the mutation rates introduced by the rest of three gRNAs were increasing more rapidly before dome stage than after dome stage, which reached over 50% of the highest rates detected at 48 hpf, indicating that the majority of genome editing events introduced by CRISPR/Cas9 occurred before dome stage, which was consistent with a recent study in zebrafish [8]. However, for different gRNAs, genome editing initiated at different stages. We evaluated sequence feature and gRNA stability of these gRNAs via SSC [13] and CRISPRscan [14], respectively. However, we did not find systematic variance between these gRNAs on sequence feature calculated by SSC, nor on gRNA stability calculated by CRISPRscan, indicating that unknown factors may trigger the initiation of CRISPR/Cas9 editing.

### Nucleosome organization does not show clear correlation with CRISPR/Cas9 efficiency

**Fig 2:**
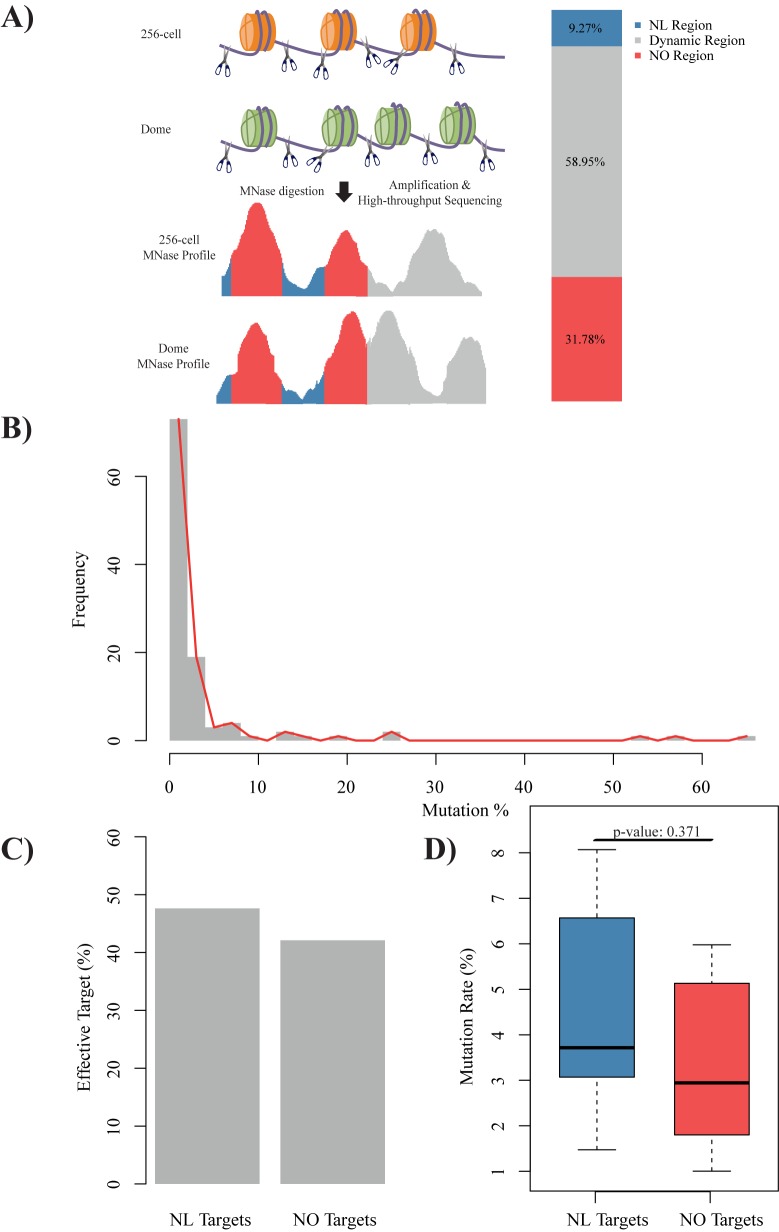
CRISPR/Cas9 efficiency did not show clear correlation with nucleosome organization. **(A)** Schematic diagram of nucleosome organization status identification according to our previous MNase-seq data (GSE44269, see Material and Method section for details). Zebrafish genebody (Refseq annotation) was divided into nucleosome linker (NL, blue) region, nucleosome organization (NO, red) region, and dynamic nucleosome region (grey). **(B)** Distribution of mutation rate. **(C)** Barplot of proportion of effective gRNA targeting on NL region (left) and NO region (right), odds ratio: 1.22. **(D)** Boxplot of mutation rate of gRNA targeting on NL region (blue) and NO region (red), t-test p-value: 0.371.

Because the majority of mutation events caused by CRISPR/Cas9 occurred before the dome stage, we hypothesized that the local chromatin structures might affect CRISPR/Cas9 efficiency mainly before the dome stage. However, our previous study showed that nucleosome organization changed dramatically at 256-cell stage and dome stage in zebrafish [20], which increases the difficulty of selecting the correct stage for evaluating the effects. To evaluate the influence of nucleosome dynamics in zebrafish embryogenesis, we combined our previously published nucleosome profiles for the 256-cell and dome stages using GeneTrack to identify consistent nucleosome occupied regions and linker regions on the gene bodies (Fig 2A; see Materials and Methods for details). As a result, the zebrafish exome was classified into three categories: 31.8% were classified as consistent nucleosome occupied regions before the dome stage, 9.3% as consistent nucleosome linker regions, and the remainder as nucleosome dynamic regions.

To evaluate the effects of nucleosome organization on CRISPR/Cas9 efficiency, we designed 110 gRNAs with low off-target potential in zebrafish (see Materials and Methods for details and S4 File for target information). We divided these targets into 16 groups and co-injected 6 or 7 gRNAs and the Cas9 mRNA into one-cell stage embryos in one experiment. Subsequently, we detected the mutation rate of each target by high-throughput sequencing, which was used to represent the CRISPR/Cas9 efficiency in this study (Fig 2B; see Materials and Methods for details). The distribution of mutation rates in our study was generally lower than that of previous reports and even more than half of the target sites were hardly mutated (defined as CRISPR/Cas9 efficiency, i.e. mutation rate, lower than 1%), which was mainly because of the low concentration of gRNA and Cas9 mRNA used for the co-injection. In total, 38 gRNAs were regarded as targeting nucleosome occupied regions (termed as group NO) and 21 were associated with consistent nucleosome linker regions (termed as group NL). For the gRNAs in the NO group, 42.1% of their targets were effective (defined as CRISPR/Cas9 efficiency higher than 1%); for the gRNAs in the NL group, the percentage was higher (47.6%, odds ratio: 1.22; Fig 2C). For the effective gRNAs in our experiments, although the gRNAs in the NO group showed lower mutation rates than those in the NL groups, the difference was not statistically significant (Fig 2D; p-value: 0.371). To confirm our observations, another independent large-scale dataset [14] was reanalyzed. Similar to the observation in our dataset, gRNAs in the NL groups had more effective targets than those in NO groups (38.8% versus 33.8%, odds ratio: 1.24; S2A Fig). However, for the effective gRNAs in that dataset, we did not observe the difference of mutation rates between the gRNAs in the NO group and those in the NL groups (S2B Fig; p-value: 0.225). In summary, our results did not show a significant correlation between nucleosome organization and CRISPR/Cas9 efficiency, which is consistent to a previous study [14].

### Chromatin accessibility is positively correlated with CRISPR/Cas9 efficiency

**Fig 3:**
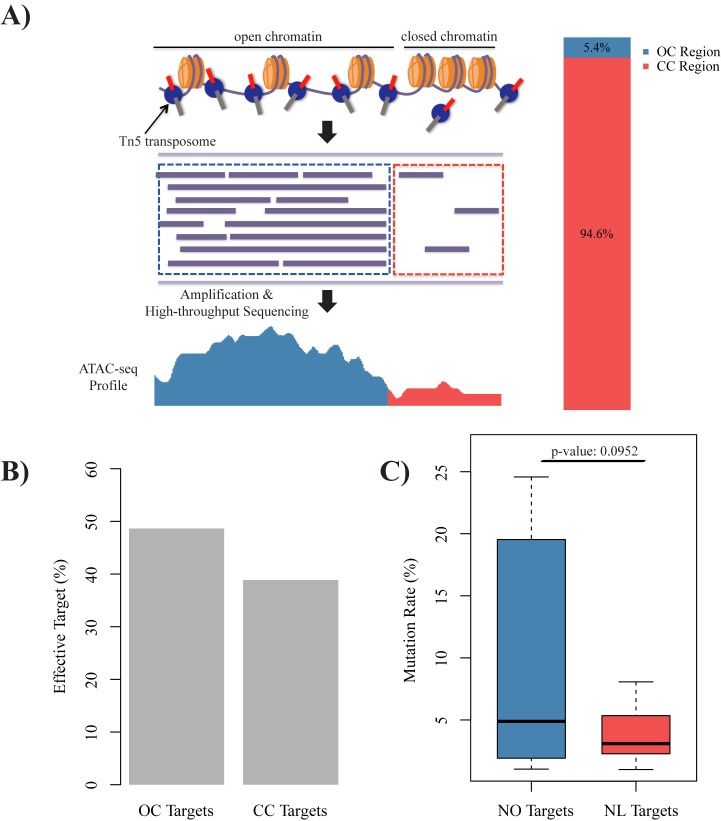
CRISPR/Cas9 efficiency is positively correlated with chromatin accessibility. **(A)** Schematic diagram of chromatin accessibility status identification according to our ATAC-seq data (see Material and Method section for details). Zebrafish genome was divided into open chromatin (OC, blue) region and close chromatin (CC, red) region. **(B)** Barplot of proportion of effective gRNA targeting on OC region (left) and CC region (right), odds ratio: 1.52. **(C)** Boxplot of mutation rate of gRNA targeting on OC region (blue) and CC region (red), t-test p-value: 0.0952.

Chromatin accessibility influences the interactions of *trans*-acting factors and *cis*-acting DNA elements. To evaluate the effects of chromatin accessibility on CRISPR/Cas9 efficiency, we generated ATAC-seq data from the 256-cell and dome stages (see Materials and Methods for details). When comparing the ATAC-seq peaks (open chromatin regions) at the two stages, we observed that most open chromatin regions detected in the 256-cell stage were also observed in the dome stage (S3A Fig), which indicated that the open chromatin regions may gradually increase before the dome stage in zebrafish. In this study, we combined open regions predicted by ATAC-seq at the 256-cell and dome stages and identified 5.4% of the zebrafish genome as open chromatin regions (Fig 3A; see Materials and Methods for details). For the gRNAs designed in this study, 37 were classified as targeting open chromatin regions (termed as group OC) and 73 were associated with closed chromatin regions (termed as group CC). The targets were effective for 38.9% of the gRNAs in the CC group, whereas the targets were effective for 48.6% of the gRNAs in the OC group (Fig 3B; odd ratio: 1.52). Moreover, the mutation rates of targets for the OC group were higher than those for the CC group (Fig 3C; p-value: 0.0952), which indicated that chromatin accessibility might have a role in controlling the interaction of Cas9 and the PAM motif. We also confirmed our observations by reanalyzing another independent dataset (S3B Fig, odds ratio: 1.45; S3C Fig, p-value: 0.0187). Taken together, our results showed that chromatin accessibility had clear positive correlation effecting on CRISPR/Cas9 efficiency in zebrafish.

### Chromatin accessibility information can be applied to improve gRNA designs

**Fig 4:**
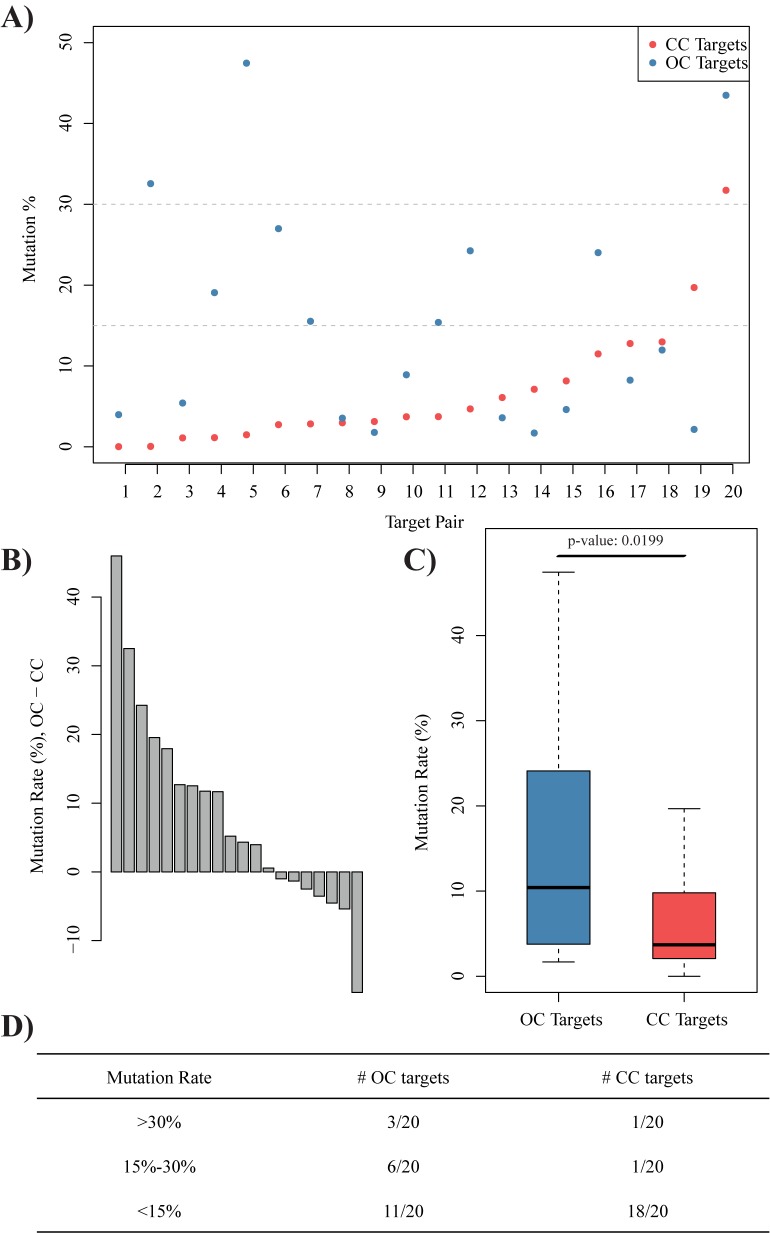
Paired co-injection experiments indicated chromatin accessibility information could be applied to improve gRNA designs. **(A)** Dot plot of mutation rates for gRNAs targeting on OC regions (blue), and gRNAs targeting on CC regions (red). **(B)** Barplot of the mutation rate differences between paired co-injected gRNAs. **(C)** Boxplot of mutation rates of gRNA targeting on OC region (blue) and CC region (red), paired t-test p-value: 0.0199. **(D)** Summary of discretized mutation rates for gRNAs targeting on OC regions and CC regions separately.

Although our results showed that chromatin accessibility had a clear positive correlation with CRISPR/Cas9 efficiency, the efficiencies detected were generally too low to be practical in zebrafish mutant construction. To figure out whether the co-injection of 6 or 7 gRNAs led to the low CRISPR/Cas9 efficiency, and to confirm the effects of chromatin accessibility on CRISPR/Cas9 efficiency, we re-designed 40 gRNAs with low off-target potential in zebrafish (See S5 File for target information). We divided these targets into 20 groups and co-injected a pair of gRNAs and the Cas9 mRNA into one-cell stage embryos in one experiment. It should be noticed that, in each experiment both gRNAs showed similar sequence feature score calculated by SSC [13], while one gRNA was designed to target OC region and another was designed to target CC region. We detected the CRISPR/Cas9 efficiency for each designed gRNA by high-throughput sequencing, and we indeed observed much higher mutation rates than those from co-injection of 6 or 7 gRNAs (mean value: 14.8% versus 3.9%; percentage of effective targets: 96.1% versus 42.2%). For co-injected gRNA pairs, the mutation rates of targets for OC regions were generally higher than those for CC regions (Fig 4A–4C; p-value: 0.0199). Furthermore, 9 out of 20 targets for OC regions showed mutation rates higher than 15%, including 3 higher than 30%, while only 2 out of 20 targets for CC regions showed mutation rates higher than 15% (Fig 4D), indicating that the chromatin accessibility information could be applied to benefit the zebrafish mutant construction. Similarly, we also performed the co-injection of paired re-designed gRNAs based on nucleosome organization information (See S6 File for target information). The mutation rates of targets for NL regions and NO regions did not show significant difference (S4 Fig), which is consistent to our above results. In summary, our results indicated the integration of chromatin accessibility information could improve gRNA design in zebrafish.

**Fig 5:**
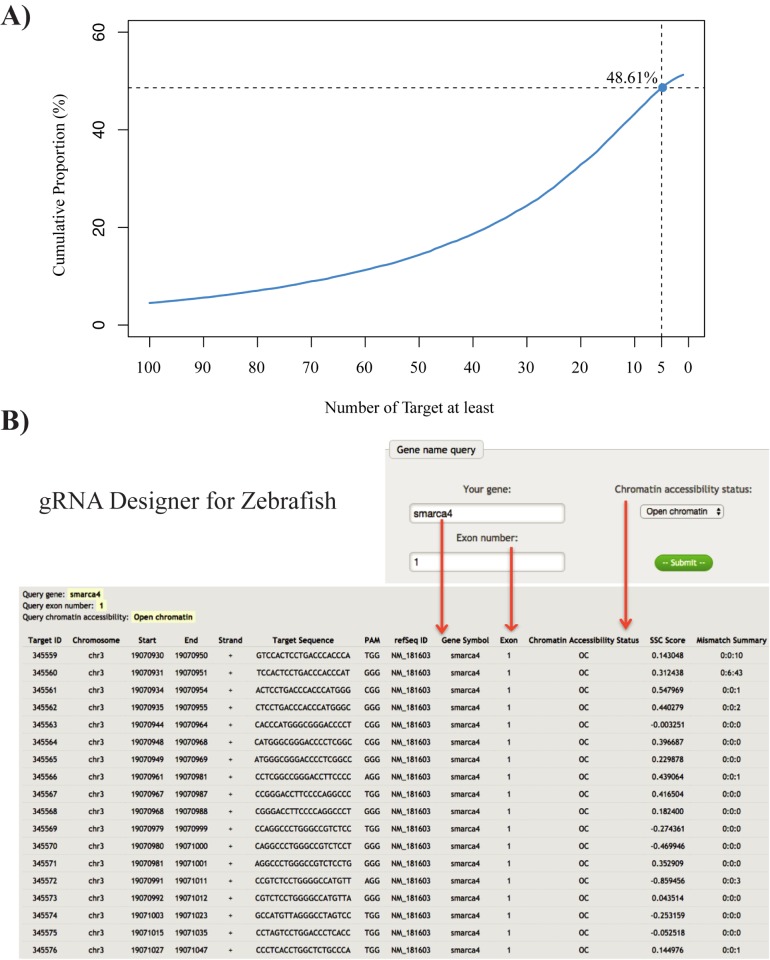
A webserver was developed to improve CRISPR/Cas9 target design. **(A)** Cumulative proportion curve of number of genes (Refseq annotation) on which there are at least 1–100 potential gRNA targets on OC region (blue). 48.61% of genes have at least 5 targets on OC region. **(B)** Screenshot of a query example for gene *smarca4* exon 1 on OC regions at the online webserver.

To evaluate the practicability to incorporate chromatin accessibility information, we first scanned the whole zebrafish exome and obtained 2,922,312 potential gRNA targets in zebrafish (see Materials and Methods for details). Among these, using chromatin accessibility information, we found that 336,022 (11.5%) candidate gRNA targets were located in OC regions, which presented potentially higher CRISPR/Cas9 efficiency for the corresponding gRNAs. Totally 48.61% of all zebrafish genes contained five or more candidate gRNA targets in OC regions (Fig 5A). Taken together, there are sufficient candidate gRNA targets with chromatin accessibility features favoring CRISPR/Cas9 editing are available for around half of zebrafish genes. To facilitate the exploitation of chromatin accessibility information in gRNA designs by the zebrafish community, we developed a webserver named gRNA Designer for Zebrafish to integrate candidate gRNAs targeting zebrafish exons with chromatin accessibility information (http://compbio.tongji.edu.cn/crispr; Fig 5B). For each gene, all candidate gRNAs targeting exons were listed, and the following features were included for each candidate: 1) target classifications based on chromatin accessibility using “OC” or “CC” labels; 2) evaluations of gRNA sequence scores calculated by SSC [13]; and 3) specificity evaluations of gRNA calculated according to 0–2 potential mismatched sites on the zebrafish genome (see Materials and Methods for details). Users can easily select suitable gRNAs for genome editing from the query output results.

## Discussion

Compared with genome editing experiments in cell lines, the purpose of genome editing in model organisms is to obtain stable heritable progeny (homozygous mutants) from the founder generation. Therefore, researchers should care more about success rates, or mutation rates, when they design editing targets in model organisms because experimental materials as well as time and labor costs are important considerations in model organism experiments. In cell lines, for mutation events generated with varying degrees of Cas9 involvement, mutated sequences can easily be maintained by cell line amplification. However, in model organisms, parents with sufficiently high mutation rates must be identified to obtain mutated progeny, or the earlier work to establish editing operations may have been conducted in vain. In our study, we chose zebrafish as a model organism to investigate the CRISPR/Cas9 system. Experiments in zebrafish differ from experiments in mice because of the difference in the time course of embryogenesis. For instance, the 1 k-cell stage is reached in 3 hpf in zebrafish. Therefore, injected gRNA and Cas9 mRNA will easily dilute and even resolve during the high-speed mitosis of embryos. However, embryogenesis process in murine models is relatively slower (more than 24 h to achieve the 2-cell embryo stage) than that in zebrafish, which may lead to different mutation results introduced by gRNA and Cas9 mRNA. Generally, germ line cells mutated during embryogenesis are more likely to generate mutant progeny [8]. However, few germ line cells are observed in the various stages in zebrafish, even in the dome stage. Therefore, optimizing Cas9 target selection may provide significant and meaningful improvements to the generation of mutation events and homozygous mutants. In sum, CRISPR/Cas9 efficiency, which determines the mutation rate, has a greater effect in genome editing design based on model organisms, especially zebrafish, than on cell lines. Moreover, the conclusions based on zebrafish in this study are applicable to genome editing in other models via the CRISPR/Cas9 system.

Because the local chromatin structure is highly dynamic during embryogenesis in zebrafish, we first identified the main working period of CRISPR/Cas9. Our results indicated that CRISPR/Cas9 mainly works in zebrafish embryos before the dome stage, which is consistent with the results of a previous study [8]. However, another study claimed that Cas9 digests target sequences at 6 hours after injection [28]. In the study from Sung *et al*., T7EI was used to detect mutations caused by Cas9. Despite the different methods (we detected mutation rates via RE), we still regarded the difference as reasonable results because the injection of gRNA/Cas9 mixture into the one-cell-embryo would slow down the embryogenesis process. Usually, we found that the zebrafish embryos could not reach dome stage at exact 4.3 hours after injection. However, we collected dome-stage embryos mainly depended on embryo morphology rather than the time post injection. Thus, we obtained dome-stage embryos later than 4.3 hpf in our study, indicating that our observation was close to the results from Sung *et al*. Therefore, as mentioned above, the effects of CRISPR/Cas9 in zebrafish embryo may be determined according to the cell division times. Because of the restricted experimental conditions in stages earlier than the 64-cell stage and other factors, the exact time at which Cas9 exerts an effect is difficult to determine. However, the finding that Cas9 primarily generates mutations before the dome stage is meaningful for experiment designs of zebrafish models.

Our results indicated nucleosome organization did not show clear correlation with CRISPR/Cas9 efficiency in zebrafish. In two recent studies in mammalian cell lines, nucleosome organization was shown to have effects on CRISPR/Cas9 efficiency [16, 17], which is inconsistent to our study and a previous study in zebrafish [14]. A potential reason for the inconsistence is that nucleosome organization is highly dynamic during zebrafish early embryogenesis, as we shown in our previous study [20], which is distinct to the features of nucleosome organization in mammalian cell lines. In another word, NL regions at both 256-cell and dome stages may become NO state at another stages, for example 512-cell stage. To figure out the inconsistence above, nucleosome profiles at more stages of zebrafish embryogenesis are required. Different from the case of nucleosome organization, chromatin accessibility was correlated with both effectiveness of gRNAs and CRISPR/Cas9 efficiency in zebrafish. As we observed in this study, chromatin accessibility may be gradually established during zebrafish early embryogenesis, which means regions opened at 256-cell stage are very likely to keep the openness at later stages. Our study took advantage of this feature of chromatin accessibility to improve the CRISPR/Cas9 efficiency in zebrafish, and our results also indicated that Cas9 prefers to work at relatively open chromatin regions in zebrafish early embryogenesis.

Although this study mainly focused on the correlation of local chromatin structures and CRISPR/Cas9 efficiency, we also acknowledge the effects of sequence features on effective gRNA selection. Previous studies have shown the effects of sequence features on nucleosome organization in yeast [29–33], thereby indicating that some local chromatin structure information may not be independent of the sequence features of effective gRNA. However, our previous study in zebrafish did not observe clear effects of sequence features on nucleosome organization [20]. In zebrafish, we classified all potential gRNA targets located on exons based on their local chromatin structure features and compared the distributions of sequence feature scores for each group. Significant differences were not observed among classes (S5 Fig), which indicate that nucleosome organization or chromatin accessibility information is largely independent of sequence features related to the effectiveness. For each potential gRNA located in zebrafish gene exons, both the sequence feature score and chromatin accessibility features were displayed in our webserver gRNA Designer for Zebrafish, which provides the users the possibility of combining both sequence features and local chromatin structure features to accelerate homozygous mutant construction in zebrafish.

## Conclusion

In summary, in this study we first identified that the CRISPR/Cas9 system mainly generates mutation before the dome stage in zebrafish. Subsequently, we found that chromatin accessibility is positively correlated with CRISPR/Cas9 efficiency. However, nucleosome organization status does not show clear correlation with CRISPR/Cas9 efficiency. Therefore, we established an online database, which contains all potential CRISPR/Cas9 targets in zebrafish genome to facilitate the zebrafish community to take advantage of local chromatin structure information.

## Supporting information

S1 Fig CRISPR/Cas9 efficiencies vary greatly for different gRNAs. **(A)** Distribution of maximum difference of Cas9 efficiency on same gene (data from paper of Moreno-Mateos *et al*.). **(B)** Distribution of gRNA efficiency (data from CRISPRz). **(C)** Boxplot of SSC score of ineffective gRNAs (red) and effective gRNAs (blue). **(D)** Scatterplot of mutation ratio and target SSC score.
(EPS)

S2 Fig CRISPR/Cas9 efficiency did not show clear correlation with nucleosome organization (data from paper of Moreno-Mateos *et al*.). **(A)** Barplot of proportion of effective gRNA targeting on NL region (left) and NO region (right), odds ratio: 1.24. **(B)** Boxplot of mutation ratio of gRNA targeting on NL region (blue) and NO region (red), t- test p-value: 0.225.
(EPS)

S3 Fig CRISPR/Cas9 efficiency is positively correlated with chromatin accessibility. **(A)** Venn diagram of 256-cell stage and dome stage ATAC-seq peaks number. **(B)** Barplot of proportion of effective gRNA targeting on OC region (left) and CC region (right), odds ratio: 1.45. **(C)** Boxplot of mutation ratio of gRNA targeting on OC region (blue) and CC region (red), t-test p-value: 0.0187. **(D)** Barplot of proportion of effective gRNA targeting on on ‘NL and OC’ region, ‘NO and OC’ region, ‘NL and CC’ region and ‘NO and CC’ region.
(EPS)

S4 Fig Paired co-injection experiments indicated that nucleosome organization information was useless to improve gRNA designs. **(A)** Dot plot of mutation rates for gRNAs targeting on NL regions (blue) and gRNAs targeting on NO regions (red). **(B)** Barplot of the mutation rate differences between paired co-injected gRNAs. **(C)** Boxplot of mutation rates of gRNA targeting on on NL region (blue) and NO region (red), paired t-test p-value: 0.803. **(D)** Summary of discretized mutation rates for gRNAs targeting on NL regions and NO regions separately.
(EPS)

S5 Fig Sequence feature and local chromatin structure are two distinct independent features. **(A)** Boxplot of SSC score in NL group targets (blue) and NO group targets (red). **(B)** Boxplot of SSC score in OC group targets (blue) and CC group targets (red).
(EPS)

S1 File BWA mapping summary of each target involved. (XLS)

S2 File Summary of the ATAC-seq data. (XLS)

S3 File Targets information summary of time-course experiment design. (XLS)

S4 File Targets information summary of 110 targets design. (XLS)

S5 File Targets information summary of OC/CC target pairs design. (XLS)

S6 File Targets information summary of NL/NO target pair design. (XLS)
